# Risk factors and prediction model of nomogram for preoperative calf muscle vein thrombosis in geriatric hip fracture patients

**DOI:** 10.3389/fmed.2023.1236451

**Published:** 2023-09-01

**Authors:** Jiabao Jiang, Fei Xing, Rong Luo, Zhao Chen, Hao Liu, Zhou Xiang, Xin Duan

**Affiliations:** ^1^Department of Orthopedic Surgery, Orthopedic Research Institute, West China Hospital, Sichuan University, Chengdu, Sichuan, China; ^2^Department of Orthopedics Surgery, West China Sanya Hospital, Sichuan University, Sanya, Hainan, China; ^3^Department of Orthopedic Surgery, The Fifth People's Hospital of Sichuan Province, Chengdu, Sichuan, China

**Keywords:** calf muscular vein thrombosis, deep vein thrombosis, geriatric hip fracture, risk factors, nomogram

## Abstract

**Introduction:**

Calf muscular vein thrombosis (CMVT) is a common complication in geriatric hip fracture patients. Despite its high incidence, prior research on the topic is limited. The occurrence of CMVT in patients will prolong the preoperative waiting time and even lead to serious thromboembolic events, which can be detrimental to the patient’s prognosis. Therefore, this study aimed to identify the risk factors for preoperative CMVT in geriatric hip fracture patients and construct a nomogram model to predict the risk of preoperative CMVT in patients.

**Materials and methods:**

Geriatric hip fracture patients who underwent surgery between January 2019 and January 2022 were included. The patients were categorized into two groups depending on whether they had preoperative CMVT, confirmed through Color Doppler ultrasound or venography examination. Univariate and multivariate logistic regression analyses were used to analyze demographic characteristics, medical history, comorbidities, and laboratory tests. A nomogram was constructed to predict preoperative CMVT in geriatric hip fracture patients based on the results of the multivariate logistic regression.

**Results:**

Three hundred and eighty-eight geriatric hip fracture patients, including one hundred and thirty-four patients with CMVT and two hundred and fifty-four patients without CMVT, were ultimately included in our study. After multivariable logistic regression analysis, the time from injury to admission, smoking history, serum albumin levels, and D-dimer levels was identified as independent risk factors and was entered into a nomogram model. The nomogram showed robust discrimination, with an area under the receiver operating characteristic curve of 0.805. The calibration curve showed strong agreement between the CMVT probabilities predicted by the nomogram and the actual probabilities. The decision curve analysis illustrates the excellent clinical utility of the model.

**Conclusion:**

We have constructed a new nomogram prediction model that can effectively predict the risk of preoperative CMVT in geriatric hip fracture patients based on their medical history and blood test results. This model can help clinicians make individualized predictions of CMVT that are tailored to each patient’s unique circumstances.

## 1. Introduction

With the accelerated aging of the global population, the incidence of geriatric hip fractures is gradually increasing and has become a serious worldwide public health problem. In 1996, it was estimated that there were approximately 1.7 million new cases of hip fracture in the geriatric population worldwide, and it is expected that there will be as many as 6.3 million new cases worldwide by 2050 (1). The impact of hip fractures on the geriatric population is considerable, including increased mortality, financial burden, and reduced mobility and quality of life (2). For most of these patients, surgery is the preferred treatment for a positive prognosis, but there are potential risks and complications associated with surgical treatment.

Calf muscle vein thrombosis (CMVT) is a subtype of distal deep vein thrombosis (DVT) in which the thrombosis is confined to the venous plexus of the soleus and gastrocnemius muscles (3). Despite a high incidence, CMVT often presents with insidious symptoms and is frequently overlooked by clinicians (4). Recent studies have shown that if CMVT is not properly treated and managed, approximately 20% of patients may progress proximally into the main veins to form a full-limb venous thrombosis, resulting in serious complications such as thrombus dislodgement and even life-threatening pulmonary embolism (5, 6). Several risk factors have been associated with DVT formation in geriatric hip fracture patients, including advanced age, prolonged bed rest, and lower limb swelling (7). CMVT is the most common type of DVT in geriatric hip fracture patients, and preoperative CMVT may lead to delayed surgery, significantly increased postoperative mortality and complication rates, and adverse effects on the patient’s prognosis (8, 9). Therefore, it is necessary to develop a validated model to predict the risk of preoperative CMVT in geriatric hip fracture patients.

The nomogram has been recognized as a reliable tool to create a simple and intuitive statistical predictive model to quantify the risk of clinical events (10). In this retrospective study, we aimed to identify the risk factors associated with preoperative CMVT in geriatric hip fracture patients to accurately predict these patients. We subsequently constructed a nomogram to predict the potential risk of CMVT, providing clinicians with a personalized decision tool aimed at reducing the incidence of this serious disease.

## 2. Materials and methods

### 2.1. Patients

This research received approval from the Ethical Review Committee of West China Hospital, Sichuan University. The study participants were geriatric individuals who suffered hip fractures and underwent surgical interventions at West China Hospital from January 2019 to January 2022. The researchers adhered to the Strengthening the Reporting of Observational Studies in Epidemiology (STROBE) reporting guidelines to ensure accuracy and transparency in the study design and reporting (11). The inclusion criteria for our study were as follows: (1) patients aged 65 years or older; (2) acute hip fractures including femoral neck and intertrochanteric fractures that occurred within 7 days; (3) fractures confirmed by preoperative X-ray or CT; and (4) patients who underwent surgical intervention, including joint replacement and internal fixation. The exclusion criteria for our study were as follows: (1) multiple fractures, open fractures, or pathological fractures; (2) fractures accompanied by vascular or nerve lesions; (3) history of hip surgery for any reason; (4) history of venous thromboembolism or anticoagulation therapy (e.g., aspirin, clopidogrel, low molecular heparin, or other drugs) within the 3 months before the fracture; (5) coexistence of DVT at other locations than CMVT; and (6) incomplete clinical data.

### 2.2. Diagnostic criteria and prophylaxis for CMVT

**Figure 1:**
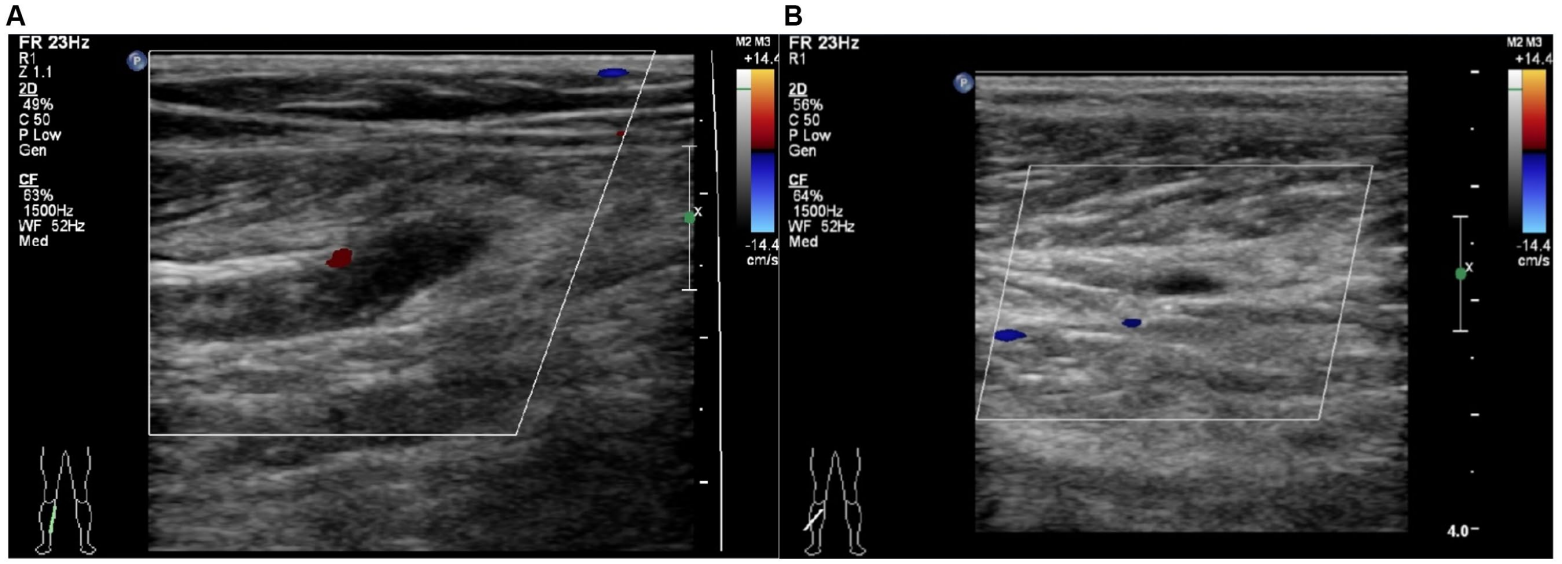
Typical image for the diagnosis of calf muscular vein thrombosis by Color Doppler ultrasound. **(A)** Right partial calf muscular vein with thickened vessel diameter and weak echogenic signal filling in the vessel cavity without obvious blood flow signal. **(B)** Right partial calf muscular vein with weak echogenic signal filling in the vessel cavity and blood flow signal filling deficit.

All patients routinely underwent Color Doppler ultrasound for thrombosis screening on admission. The diagnostic criteria for fresh thrombosis are solid echogenicity in the vessel and absence of blood flow signal or a constant intraluminal filling defect (Figure 1) (12). The Color Doppler ultrasound is conducted by an experienced sonographer in the ultrasound room. The results of all ultrasound tests were scrutinized by a senior sonographer, while any differing opinions were resolved through a re-examination of the ultrasound. All patients were reviewed for ultrasound every 3 days after admission. Venography was performed if the vessels were poorly visualized by ultrasound. If a patient develops signs and symptoms of DVT during hospitalization, repeat venography or ultrasound is performed. Signs and symptoms to look out for include changes in skin temperature and color, pain, and tenderness in the calf or thigh, swelling, and a positive Homans’ or Neuhof’s sign. According to guideline recommendations all geriatric hip fracture patients at our institution received DVT prophylaxis (13). The thromboprophylaxis regimen was implemented for each patient after admission, which included the use of elastic compression stockings, intermittent pneumatic compression, and chemoprophylaxis. The chemoprophylaxis regimen consisted of administering low molecular heparin (4,000 AxaIU/0.4 mL) or fondaparinux (2.5 mg) subcutaneously once daily. Patients diagnosed with DVT received anticoagulation and thrombolytic therapy.

### 2.3. Data collection

The medical database of our hospital was used to retrospectively retrieve data on selected patients. The case data collection was completed by several clinicians who had received standardized training. The information collected included demographic characteristics, such as age, gender, body mass index (BMI), time from injury to admission, type of fracture, injury side, preoperative waiting time in hospital, medical history (including smoking, drinking, cerebrovascular disease, and malignancy), comorbidities (including hypertension, chronic obstructive pulmonary disease, diabetes, arrhythmia, coronary heart disease, Parkinson’s disease, renal dysfunction, liver dysfunction, and varicose veins), as well as laboratory tests such as hemoglobin, blood platelet, serum albumin, serum potassium, serum sodium, prothrombin time (PT), activated partial prothrombin time (APTT), thrombin time (TT), fibrinogen, and D-dimer. This comprehensive approach was taken to ensure that all relevant data points were captured for each patient to conduct an accurate and detailed analysis of their medical history and current condition.

### 2.4. Statistical analysis

A univariate analysis was initially conducted, dividing patients into two groups: with and without CMVT. Comparisons were made between the two groups to identify significant differences in independent variables. All variables with *p* < 0.05 from the univariate analyses were included in the multivariate logistics regression model to determine the independent risk factors of CMVT. The strength of the correlation was indicated using the odds ratio (OR) with a 95% confidence interval (CI). To test the fitness of the final model, the Hosmer-Lemeshow test was performed, with results of *p* > 0.05 indicating acceptable accuracy. Based on the results from the final regression analysis, a nomogram for CMVT probability was constructed. The performance of the nomogram was assessed through discrimination and calibration. Discriminative ability was determined by calculating the area under the curve (AUC) of receiver operating characteristic (ROC) analysis. Calibration was assessed with a visual calibration plot comparing predicted and actual CMVT probabilities. Decision curve analysis was performed to assess the clinical utility of the nomogram (14). Mean and standard deviation (SD) were used to express continuous variables, while categorical variables were presented as absolute numbers and percentages. Categorical variables were compared using the χ2 analysis or Fisher’s exact test, while independent-sample *t*-tests or Wilcoxon rank sum tests were used for normally and non-normally distributed continuous variables, respectively. Statistical analyses and graphics were performed using the SPSS statistical software (version 25.0; IBM Corp, Armonk, NY, United States) and R software (version 3.1.2; The R Foundation for Statistical Computing, Vienna, Austria) with the RMS statistical packages. A *p*-value of less than 0.05 was considered statistically significant for all analyses, and all tests were two-tailed unless otherwise stated.

## 3. Results

### 3.1. Patients demographic and clinical characteristics

**Figure 2:**
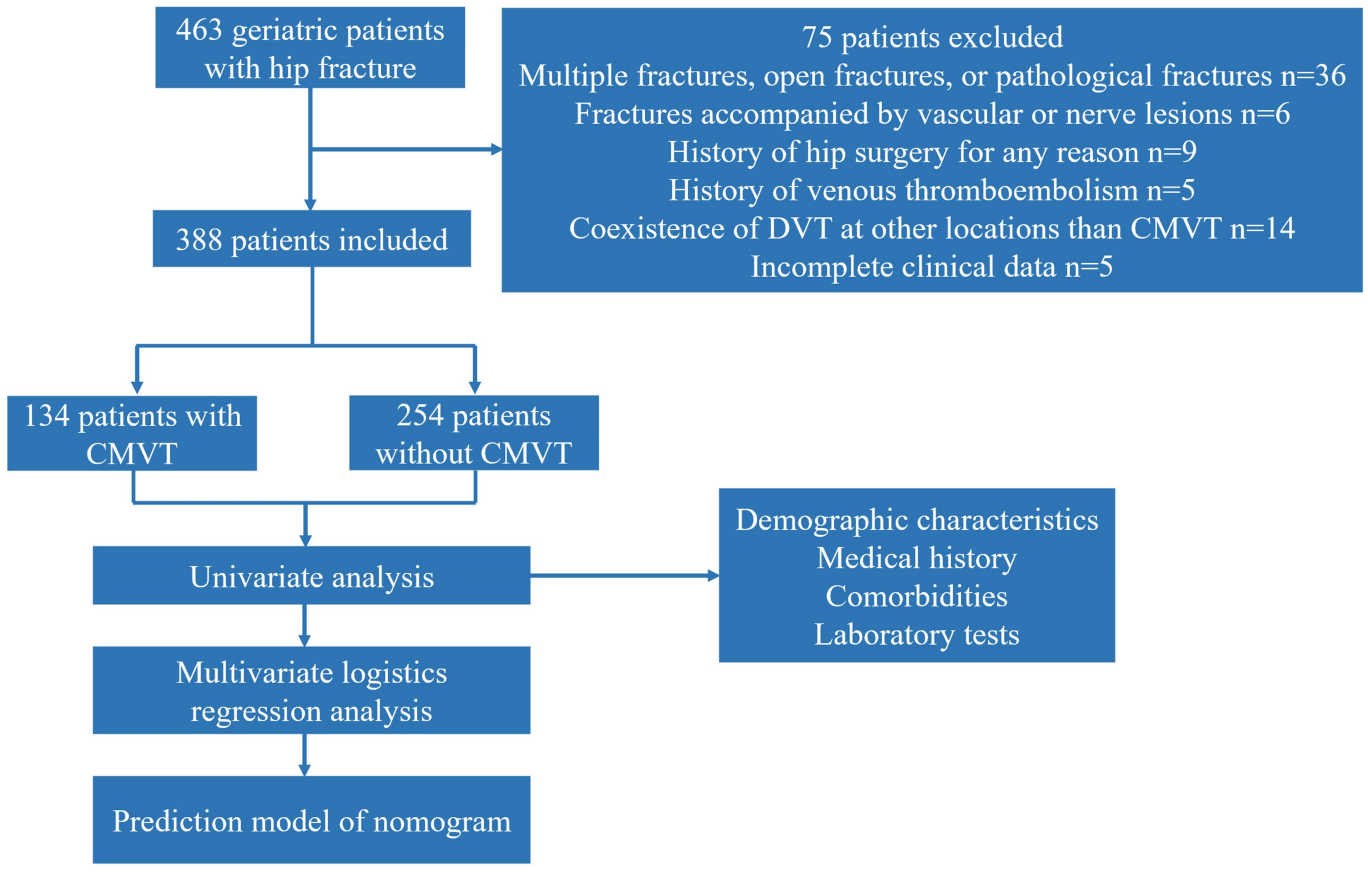
The sampling procedure used for all geriatric hip fracture patients in this study. CMVT, Calf muscular vein thrombosis.

A total of four hundred and sixty-three geriatric hip fracture patients met our inclusion criteria. Of these, thirty-six were excluded due to multiple fractures, open fractures, or pathological fractures, six were excluded due to fractures accompanied by vascular or nerve lesions, nine were excluded due to a history of hip surgery, five were excluded due to a history of venous thromboembolism, fourteen were excluded due to the coexistence of DVT at other locations than CMVT, and five were excluded due to incomplete clinical data. In summary, our study included three hundred and eighty-eight geriatric hip fracture patients, consisting of one hundred and thirty-four patients with CMVT and two hundred and fifty-four patients without CMVT. Figure 2 depicts the sampling procedure of geriatric hip fracture patients in our study. The mean age of the included patients was 80.0 ± 7.9 years and the majority of patients were female (69.8%). The demographic and clinical characteristics of the patients were shown in Table 1. Eight patients did not have CMVT on admission and were diagnosed with CMVT while waiting for surgery in the hospital. Univariate analysis was performed on the group of patients without CMVT and with CMVT. The results showed significant differences between the two groups in terms of time from injury to admission, smoking history, hemoglobin levels, platelet levels, serum-albumin levels, and D-dimer levels (*p* < 0.05). There were no significant differences between the two groups in terms of comorbidities and the remaining variables.

**Table 1:** The demographic and clinical characteristics and univariate analysis results of geriatric hip fracture patients with and without CMVT.

Variables	Total Patients (*n* = 388)	Patients without CMVT (*n* = 254)	Patients with CMVT (*n* = 134)	OR(95% CI)	*p* value*
Demographic characteristics					
Age (years)	80.0 ± 7.9	79.7 ± 8.2	80.5 ± 7.4	1.01 (0.99–1.04)	0.360
Gender					
Male	117	83 (32.7)	34 (25.4)	–	
Female	271	171 (67.3)	100 (74.6)	1.43 (0.90–2.30)	0.137
BMI	21.9 ± 3.5	21.9 ± 3.5	21.8 ± 3.6	0.99 (0.94–1.06)	0.868
Time from injury to admission (days)	2.0 ± 1.9	1.8 ± 1.7	2.5 ± 2.1	1.22 (1.10–1.37)	<0.001
Type of fracture					
Femoral neck fracture	193	130 (51.2)	63 (47.0)	–	
Intertrochanteric fracture	195	124 (48.8)	71 (53.0)	1.18 (0.78–1.80)	0.435
Injury side					
Left	194	131 (51.6)	63 (47.0)	–	
Right	194	123 (48.4)	71 (53.0)	1.20 (0.79–1.83)	0.393
Preoperative waiting time in hospital (days)	5.2 ± 3.0	5.1 ± 2.9	5.4 ± 3.2	1.04 (0.97–1.12)	0.226
Medical history					
Smoking history (current or past)					
No	264	194 (76.4)	70 (52.2)	–	
Yes	124	60 (23.6)	64 (47.8)	2.96 (1.90–4.63)	<0.001
Drinking history (current or past)					
No	303	197 (77.6)	106 (79.1)	–	
Yes	85	57 (22.4)	28 (20.9)	0.91 (0.54–1.51)	0.726
History of cerebrovascular disease					
No	325	218 (85.8)	107 (79.9)	–	
Yes	63	36 (14.2)	27 (20.1)	1.53 (0.88–2.64)	0.131
History of malignancy					
No	383	250 (98.4)	133 (99.3)	–	
Yes	5	4 (1.6)	1 (0.7)	0.47 (0.02–3.22)	0.501
Comorbidities					
Hypertension					
No	193	122 (48.0)	71 (53.0)	–	
Yes	195	132 (52.0)	63 (47.0)	0.82 (0.54–1.25)	0.354
Chronic obstructive pulmonary disease					
No	329	221 (87.0)	108 (80.6)	–	
Yes	59	33 (13.0)	26 (19.4)	1.61 (0.91–2.83)	0.096
Diabetes					
No	293	184 (72.4)	109 (81.3)	–	
Yes	95	70 (27.6)	25 (18.7)	0.60 (0.36–1.00)	0.054
Arrhythmia					
No	342	228 (89.9)	114 (85.1)	–	
Yes	46	26 (10.2)	20 (14.9)	1.54 (0.82–2.87)	0.177
Coronary heart disease					
No	330	210 (82.7)	120 (89.6)	–	
Yes	58	44 (17.3)	14 (10.4)	0.56 (0.28–1.03)	0.074
Parkinson’s disease					
No	378	249 (98.0)	129 (96.3)	–	
Yes	10	5 (2.0)	5 (3.7)	1.93 (0.53–7.06)	0.305
Renal dysfunction					
No	357	235 (92.5)	122 (91.0)	–	
Yes	31	19 (7.5)	12 (9.0)	1.22 (0.56–2.56)	0.611
Liver dysfunction					
No	378	249 (98.0)	129 (96.3)	–	
Yes	10	5 (2.0)	5 (3.7)	1.93 (0.53–7.06)	0.305
Varicose veins					
No	384	251 (98.8)	133 (99.3)	–	
Yes	4	3 (1.2)	1 (0.7)	0.63 (0.03–4.97)	0.689
Laboratory tests					
Hemoglobin(g/L)	110.4 ± 21.1	112.2 ± 20.7	107.0 ± 21.5	0.99 (0.98–1.00)	0.020
Blood platelet(10^9/L)	164.9 ± 67.6	158.0 ± 62.3	177.8 ± 75.3	1.00 (1.00–1.01)	0.007
Serum albumin(g/L)	36.9 ± 4.4	37.7 ± 4.1	35.4 ± 4.7	0.88 (0.84–0.93)	<0.001
Serum potassium(mmol/L)	3.9 ± 0.5	3.9 ± 0.4	4.0 ± 0.6	1.53 (1.00–2.36)	0.053
Serum sodium(mmol/L)	139.0 ± 3.8	138.9 ± 3.9	139.2 ± 3.7	1.02 (0.97–1.08)	0.408
PT(s)	11.6 ± 1.9	11.6 ± 2.3	11.6 ± 0.9	0.99 (0.87–1.11)	0.924
APTT(s)	28.7 ± 3.6	28.9 ± 3.6	28.4 ± 3.6	0.97 (0.91–1.03)	0.273
TT(s)	17.6 ± 7.0	17.8 ± 8.0	17.3 ± 4.4	0.99 (0.93–1.02)	0.512
Fibrinogen(g/L)	4.0 ± 1.2	4.0 ± 1.2	4.1 ± 1.3	1.11 (0.93–1.31)	0.244
D-dimer (μg/ml)	8.5 ± 8.3	7.3 ± 7.2	10.7 ± 9.5	1.05 (1.02–1.08)	<0.001

CMVT, calf muscle vein thrombosis; OR, odds ratio; CI, confidence interval. *Represents *p* < 0.05.

### 3.2. Multivariate logistic regression analysis results

**Figure 3:**
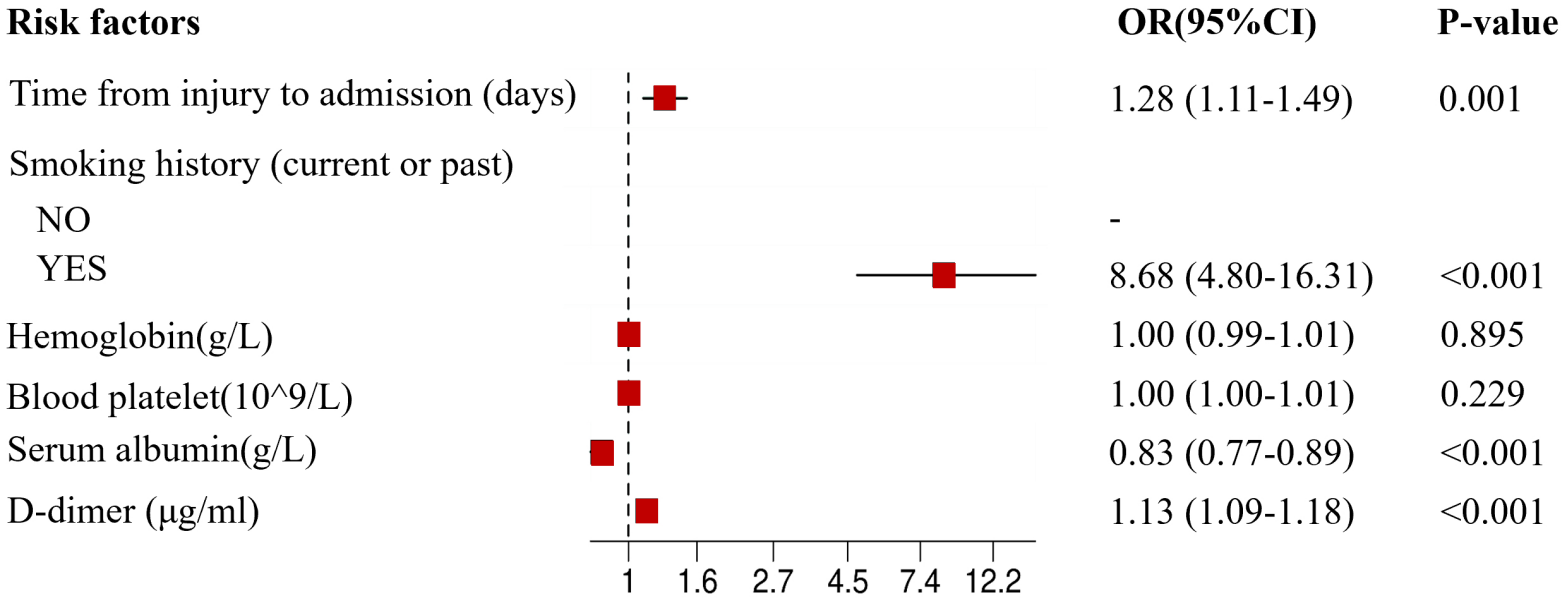
The forest plot for multivariate logistic regression analysis in geriatric hip fracture patients. OR, odds ratio; CI, confidence interval.

After univariate analysis, variables such as time from injury to admission, smoking history, hemoglobin levels, platelet levels, serum-albumin levels, and D-dimer levels were included in the multivariate logistic regression analysis. The multivariate analysis showed that the incidence of CMVT was significantly associated with time from injury to admission (*p* = 0.001), smoking history (*p* < 0.001), serum-albumin levels (*p* < 0.001), and D-dimer levels (*p* < 0.001); however, hemoglobin levels and platelet levels were not significant. To validate our findings, we performed the Homser-Lemeshow test and found that the final model had good fitness (X^2^ = 11.620, *p* = 0.169). Therefore, time from injury to admission, smoking history, serum-albumin levels, and D-dimer levels were independent risk factors for preoperative CMVT in geriatric hip fracture patients. The results of the multivariable logistic regression analysis are presented as forest plots in Figure 3. Patients in the CMVT group had longer time from injury to admission, lower serum albumin levels, and higher D-dimer levels than patients without CMVT. The detailed results of the multivariate logistic regression analysis are shown in Table 2.

**Table 2:** Multivariate logistic regression analysis of the risk of CMVT.

Variables	Coefficient regression	SE	Wald	OR	95% CI	*p*-value
Time from injury to admission (days)	0.249	0.076	10.792	1.283	1.106–1.488	0.001
Smoking history (current or past)	2.161	0.311	48.253	8.681	4.718–15.973	<0.001
Hemoglobin (g/L)	0.001	0.007	0.017	1.001	0.988–1.014	0.895
Blood platelet (10^9/L)	0.002	0.002	1.450	1.002	0.998–1.007	0.229
Serum albumin (g/L)	−0.184	0.037	24.129	0.832	0.773–0.895	<0.001
D-dimer (μg/ml)	0.127	0.020	42.055	1.135	1.092–1.179	<0.001

CMVT, calf muscle vein thrombosis; SE, standard error; OR, odds ratio; CI, confidence interval.

### 3.3. Nomogram for predicting the risk of preoperative CMVT

**Figure 4:**
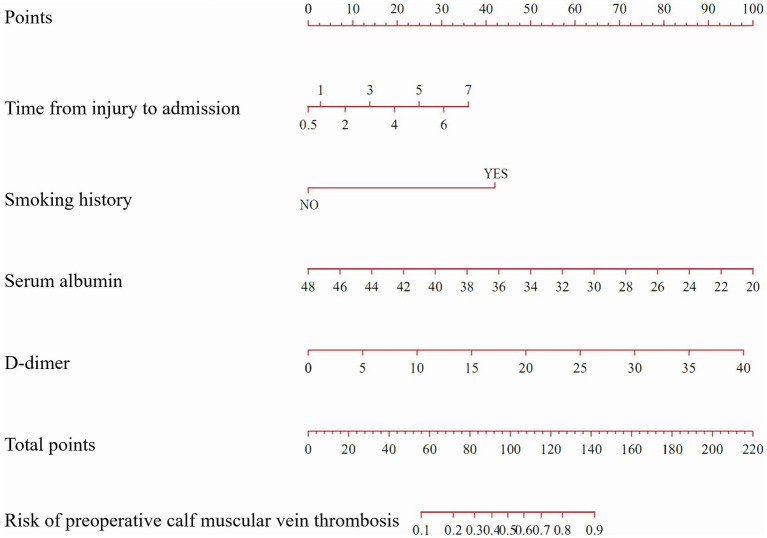
A nomogram predicting the risk of preoperative calf muscular vein thrombosis for geriatric hip fracture patients. (The nomogram assigned a specific score on the point scale axis for each variable, and these individual scores were totaled to determine the total score. This total score can be projected onto the lower total point scale to estimate the risk of preoperative calf muscular vein thrombosis for geriatric hip fracture patients).

A nomogram was constructed based on the multivariate logistic regression analysis containing four independent risk factors predicting the occurrence of CMVT preoperatively in geriatric hip fracture patients (Figure 4). Total scores were calculated using time from injury to admission, smoking history, serum albumin levels, and D-dimer levels. The values for each of these variables were scored on the topmost scaled axis. By summing each score, a total score can be easily calculated. By projecting the total score to the lower total point scale, we could estimate the risk of preoperative CMVT in geriatric hip fracture patients.

### 3.4. Performance and clinical utility of the nomogram

**Figure 5:**
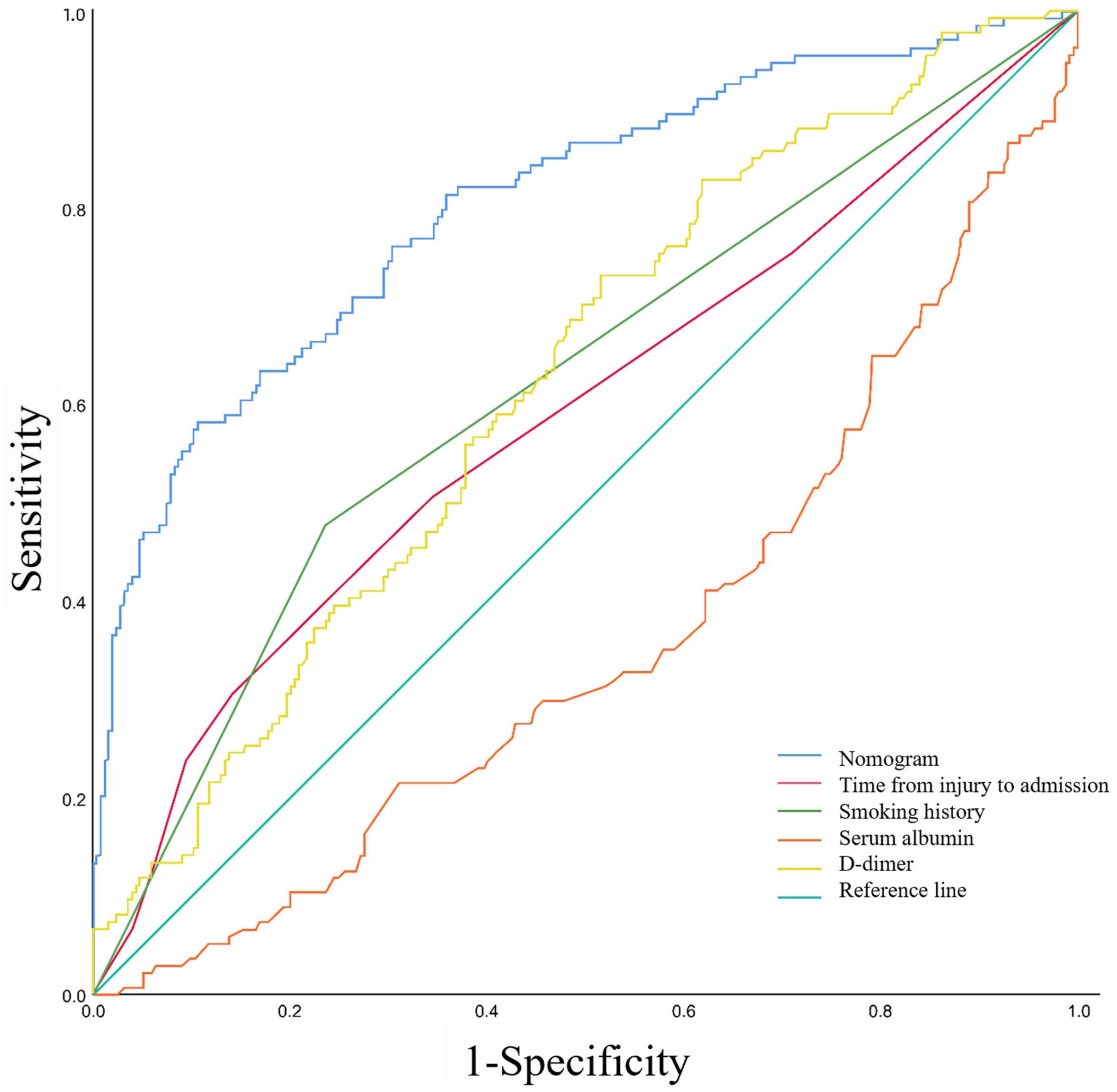
The receiver operating characteristic curves of nomogram and different risk factors.

**Figure 6:**
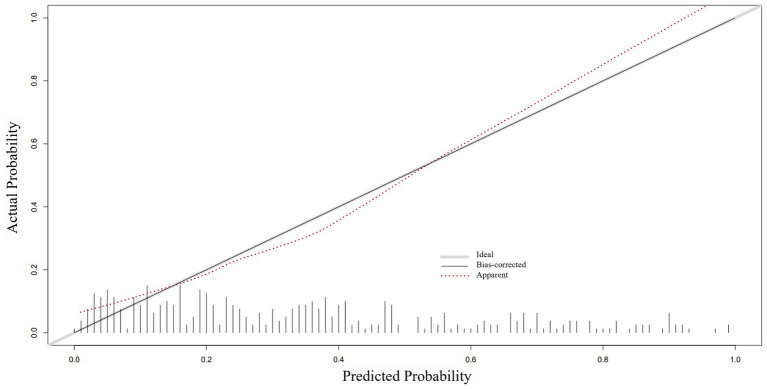
The calibration curve of the nomogram. The *x*-axis represents the risk predicted by the nomogram. The *y*-axis represents the patients diagnosed with preoperative calf muscular vein thrombosis. The diagonal light grey thick line indicates the perfect prediction of the ideal model. The apparent line represents the performance of the nomogram.

**Figure 7:**
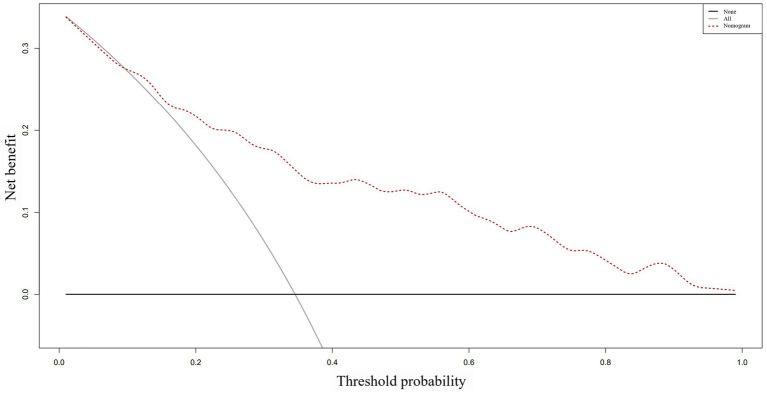
The decision curve analysis of the nomogram. The *y*-axis represents the net benefit, while the dotted line represents the nomogram. The All line assumes that all patients have preoperative calf muscular vein thrombosis, whereas the None line assumes that no patient has preoperative calf muscular vein thrombosis. The net benefit was calculated by subtracting the fraction of patients who are falsely diagnosed from the fraction who are diagnosed with the condition, and weighing this result by the relative harm of not receiving treatment compared to the negative consequences of receiving unnecessary treatment.

Based on the ROC analysis, the nomogram exhibited robust discrimination, with an AUC value of 0.805 (95% CI 0.757–0.852). The AUC values for time from injury to admission, smoking history, serum albumin levels, and D-dimer levels were 0.590, 0.621, 0.359, and 0.624, respectively. The ROC curves of the different factors were depicted in Figure 5. Meanwhile, the calibration curve of the nomogram was shown in Figure 6, indicating a strong consistency between the CMVT probabilities predicted by the nomogram and the actual probabilities. The decision curve analysis for the nomogram was presented in Figure 7. The curve demonstrates that if a patient’s threshold probability falls between 10 and 100%, utilizing the nomogram for predicting preoperative CMVT offers greater benefits compared to using either the treat-all-patients or the treat-none scheme. In our study, the incidence of preoperative CMVT in geriatric hip fracture patients was 34.5%. At a threshold of 34.5%, the decision curve was above the none and all lines, indicating that the model has clinical utility.

## 4. Discussion

CMVT has a high incidence in geriatric hip fracture patients, but it has not received much attention in the past. It was believed to dissolve or mechanize and was considered less dangerous and not life-threatening (15). However, studies have reported that isolated CMVT can directly cause pulmonary embolism (16, 17). Furthermore, patients experiencing altered hemodynamics during surgery may also experience dislodgement of CMVT, which can cause various degrees of embolization symptoms (18). Therefore, identifying risk factors for preoperative CMVT in these patients is vital to provide precise prevention or treatment to those at risk. Several studies have identified risk factors for DVT in geriatric hip fracture patients, such as age, history of malignancy, and D-dimer levels (19). However, there has been a lack of studies on the risk factors for preoperative CMVT in these patients. Our study identified that time from injury to admission, smoking history, serum albumin levels, and D-dimer levels were independent risk factors for preoperative CMVT in geriatric hip fracture patients. We have also constructed a simple and intuitive statistical predictive nomogram that can directly quantify the risk of preoperative CMVT in patients. Importantly, the model shows excellent discrimination, calibration, and clinical utility in identifying patients at high risk of preoperative CMVT, which facilitates perioperative management and treatment by clinicians.

A few studies have reported risk factors for CMVT in geriatric hip fracture patients, including smoking, time from injury to surgery, and D-dimer levels (9, 20). This is similar to the results of our study. However, previous studies have not developed predictive models for CMVT that are directly applicable to clinical practice. To our knowledge, this study is the first to construct a quantitative nomogram to predict the probability of preoperative CMVT in geriatric hip fracture patients. In our nomogram model, low serum albumin levels and high D-dimer levels were the main factors influencing the risk of preoperative CMVT, followed by smoking history and time from injury to admission.

Currently, most medical institutions use physical or pharmacological methods to prophylaxis thrombosis in geriatric hip fracture patients after the risk of bleeding has been excluded (21). However, the incidence of DVT in patients remains high during the perioperative period. It is reported that the incidence of CMVT in patients undergoing lower limb fracture surgery is as high as 60% (22). In our study, the incidence of CMVT in patients was 34.5% and most patients were asymptomatic. The majority of patients were diagnosed with CMVT on admission and only eight patients occurred CMVT during the preoperative waiting period. we believe that the majority of patients already had CMVT on admission and that a thromboprophylaxis regimen after admission can significantly reduce the incidence of CMVT. In addition, our results suggest that the longer the time from injury to admission, the higher the risk of preoperative CMVT for patients. If a patient already has CMVT at admission, it is essential to initiate thrombosis treatment instead of prophylaxis to prevent thrombus extension, acute pulmonary embolism, and recurrent thrombosis (23). Patients with preoperative CMVT were administered low molecular heparin twice daily after the risk of bleeding has been excluded in our hospital. In addition, patients were advised to brake the affected limb, and regularly reexamine Color Doppler ultrasound. If the patient’s thrombosis continues to progress, an inferior vena cava filter will be placed. Therefore, clinicians need to notice the time from injury to admission and develop individualized treatment plans for high-risk patients to prevent thromboembolic events.

There was a strong association between smoking history and patients with CMVT in our study, which is consistent with the outcomes of previous studies (20). Smoking can directly damage the intima, inhibit the production of nitric oxide and reduce vascular elasticity, thereby affecting the function of vascular endothelial cells and leading to the rupturing of vessels (24). In addition, smoking can accelerate platelet activation and aggregation, reduce fibrin degradation, increase blood viscosity, reduce blood flow, and stimulate vasoconstriction, resulting in blood prone to thrombus formation, further increasing the risk of CMVT (25). Additionally, smoking has been suggested to be associated with pulmonary embolism, malignancy, and various cardiovascular diseases and may exert a synergistic effect with thrombosis (26, 27). Overall, smoking is a significant risk factor for CMVT, and its effects on coagulation and vascular function may play a crucial role in the occurrence of CMVT. Therefore, clinicians must consider recommending smoking cessation counseling as a part of the prevention and management of CMVT.

D-dimer is a degradation product of fibrin and serves as a specific marker for the fibrinolytic process in the body (28). It can indirectly reflect the activation state of the coagulation system and enable the detection of thrombosis or other coagulation-related disorders (29). Currently, it is the most commonly used clinical indicator for the diagnosis of DVT and pulmonary embolism. Several studies on patients with lower limb fractures have found that preoperative D-dimer levels are an independent risk factor for DVT (30, 31). This was also supported by our results in the study. However, D-dimer lacks specificity, and many conditions such as infection, pregnancy, persistent blood loss, malignancy, atrial fibrillation, and other acute diseases can lead to increased D-dimer levels in patients (32). Consequently, D-dimer testing is primarily used for exclusionary diagnosis and does not suffice as the sole basis for confirming a diagnosis of DVT or pulmonary embolism, which requires a multifactorial assessment. In our study, the AUC value under the ROC curve for D-dimer levels was 0.624. Therefore, constructing a novel predictive model is necessary for identifying the risk of preoperative CMVT in geriatric hip fracture patients.

Geriatric hip fracture patients are prone to developing hypoproteinemia due to several factors. These include advanced age, reduced digestive and absorption functions, and preoperative malnutrition (33). Many patients with chronic liver and kidney disease often have compromised protein synthesis, resulting in increased protein consumption (34). Furthermore, bleeding at the fracture site causes a loss of serum albumin, particularly in patients with intertrochanteric fractures who experience more hidden blood loss (35). Our study shown that a lower serum albumin level increases the risk of CMVT. This may be attributed to the decrease in serum albumin, leading to the liver’s compensatory synthesis of albumin and other proteins (like pro-thrombotic factors such as coagulation factor V, coagulation factor VIII, and fibrinogen) (36). This results in an imbalance between pro- and anti-thrombotic factors, ultimately leading to thrombosis. Lionaki et al. (37) demonstrated that low serum albumin levels are an independent risk factor for thrombosis, and the risk of DVT increases doubly with decreasing serum albumin levels. Monitoring the patient’s serum albumin levels, improving management of comorbidities and adequate energy supplementation are recommended.

The gold standard for the diagnosis of DVT is venography, but is restricted in clinical practice due to its invasive and radioactive nature (38). Color Doppler ultrasound is currently the most used examination for the diagnosis of DVT. However, its results can be influenced by the experience of the radiologist and are challenging to perform in some primary medical institutions (39). Moreover, for patients with lower limb fractures, the position requirements during ultrasound procedures may be difficult to achieve, affecting the accuracy of results. Additionally, the waiting time for venography or ultrasound can lead to delays in the surgery, which can be detrimental to patients’ prognosis. Relevant guidelines recommend early surgery for hip fracture patients, and prolonged preoperative waiting time is associated with increased the risk of perioperative complications and mortality within 30 days postoperatively (40, 41). Therefore, we constructed a nomogram prediction model to identify patients early who are at risk of CMVT based on their medical history and blood test results. Specialist examinations can be performed on high-risk patients, reducing financial burdens on this patient population. This facilitates immediate treatment measures and reduces preoperative waiting time, benefiting the patients.

There are several limitations in this study. Firstly, we included geriatric hip fracture patients who underwent surgery; non-surgery patients were excluded. And only for prediction of CMVT. The applicability of this prediction model may be restricted. Secondly, although the internal validation of the nomogram prediction model showed excellent discrimination, calibration, and clinical utility, external validation with additional databases is required, particularly from other countries given the differences in epidemiology and clinical behavior among ethnicities. Thirdly, our study is retrospective, and potential selection bias is inevitable.

## 5. Conclusion

In conclusion, our study has identified several independent risk factors for preoperative CMVT in geriatric hip fracture patients, including the time from injury to admission, smoking history, serum albumin levels, and D-dimer levels. Additionally, we have constructed a new nomogram prediction model that can effectively predict the risk of preoperative CMVT in geriatric hip fracture patients based on their medical history and blood test results. This model provides excellent discrimination, calibration, and clinical utility, and can help clinicians make individualized predictions of CMVT that are tailored to each patient’s unique circumstances. This patient population can benefit from reducing preoperative waiting time and financial burden by effectively identifying high-risk patients.

## Data availability statement

The raw data supporting the conclusions of this article will be made available by the authors, without undue reservation.

## Ethics statement

The studies involving humans were approved by West China Hospital, Sichuan University. The studies were conducted in accordance with the local legislation and institutional requirements. Written informed consent for participation was not required from the participants or the participants’ legal guardians/next of kin in accordance with the national legislation and institutional requirements.

## Author contributions

JJ analyzed the data, drafted the manuscript, and contributed to the study design. FX, RL, ZC, and HL collected the data and assisted in the data analysis. ZX and XD designed and supervised this project. All authors contributed to the article and approved the submitted version.

## Funding

This work was supported by the National Natural Science Foundation of China (82202705), the Sino-German Center for Research Promotion (GZ1219), the Project of the Science and Technology Department of Sichuan Province (No. 2022YFS0099, No. 2023NSFSC1738) and Clinical Research Incubation project of West China Hospital of Sichuan University (2019HXFH041), Sichuan University-Luzhou Municipal People’s Government Strategic Cooperation Project (2022CDLZ-19), Sichuan Provincial Cadre Health Research Project (2023-401).

## Conflict of interest

The authors declare that the research was conducted in the absence of any commercial or financial relationships that could be construed as a potential conflict of interest.

## Publisher’s note

All claims expressed in this article are solely those of the authors and do not necessarily represent those of their affiliated organizations, or those of the publisher, the editors and the reviewers. Any product that may be evaluated in this article, or claim that may be made by its manufacturer, is not guaranteed or endorsed by the publisher.
